# Qi-Gui-Sheng-Jiang-San Decoction Regulating Hypoxia Response Through Non–Oxygen-Dependent Pathway Improves Diabetic Kidney Disease: Coupling Network Pharmacology With Experimental Verification

**DOI:** 10.1155/jdr/6399010

**Published:** 2025-11-26

**Authors:** Yi-fan Liu, Yuan-yuan Liu, Wen-yi Tian, Yao Xiao, Wei-jun Huang, Rui-xi Sun, Jie Hu, Xiao-zhe Fu, Chu-xiao Tian, Qiang Fu, Jin-xi Zhao

**Affiliations:** ^1^First Clinical Medical College & The Affiliated Hospital of Shandong University of Traditional Chinese Medicine, Shandong University of Traditional Chinese Medicine, Jinan, China; ^2^Institute of Chinese Medical Literature and Culture, Shandong University of Traditional Chinese Medicine, Jinan, China; ^3^College of Traditional Chinese Medicine, Shandong University of Traditional Chinese Medicine, Jinan, China; ^4^Nephropathy Department, Beijing University of Chinese Medicine Third Affiliated Hospital, Beijing, China; ^5^Section II of Endocrinology & Nephropathy Department, Dongzhimen Hospital Affiliated to Beijing University of Chinese Medicine, Beijing, China; ^6^Institute of Basic Theory, China Academy of Chinese Medical Sciences, Beijing, China

**Keywords:** diabetic kidney disease (DKD), HIF-1*α*, inflammation, network pharmacology, Qi-Gui-Sheng-Jiang-San (QGSJS) decoction

## Abstract

**Background:**

Diabetic kidney disease (DKD) is a major global cause of end-stage renal disease. Emerging evidence suggests that hypoxia is a critical factor in the advancement of DKD. Traditional Chinese medicine (TCM) is an effective alternative therapy for DKD. The Qi-Gui-Sheng-Jiang-San (QGSJS) decoction is an effective formula for treating DKD clinically, and its mechanism may be related to regulating hypoxia response, necessitating further investigation and a thorough analysis of the underlying biological mechanisms.

**Methods:**

Initially, we employed network pharmacology methods to collect and screen the active constituents of the QGSJS decoction from the Traditional Chinese Medicine Systems Pharmacology Database and Analysis Platform (TCMSP) and relevant chemical databases. Subsequently, the targets of these active components were predicted via the PubChem and TCMSP databases, while relevant targets associated with DKD were sourced from GeneCards, OMIM, and DrugBank. In the second phase, we built a protein–protein interaction (PPI) network via the STRING database to identify core targets. This was followed by GO and KEGG enrichment analyses to assess if the QGSJS decoction's mechanisms of action are linked to hypoxic response regulation. Finally, in vivo experiments were performed to confirm the findings from the network pharmacology analysis and to comprehensively elucidate the QGSJS decoction's mechanisms of action.

**Results:**

The network pharmacology analysis revealed 57 active components in the QGSJS decoction, capable of influencing 72 targets associated with DKD. Quercetin, kaempferol, and isorhamnetin are likely to be the key constituents of the QGSJS decoction. The PPI network suggests that HIF1A serves as a hub gene, closely associated with IL6, NFKBIA, and VEGFA. Enrichment analysis indicates that the QGSJS decoction modulates the HIF-1 signaling pathway and impacts biological processes and molecular functions linked to HIF-1*α*. In vivo studies demonstrate the QGSJS decoction's renal protective properties, suppressing the expression of HIF-1*α*, p-STAT3, p-Akt, VEGF, VEGFR, p-NF-*κ*B, and NOTCH1 in the kidneys without affecting PHD2.

**Conclusion:**

The QGSJS decoction primarily inhibits HIF-1*α* through non–oxygen-dependent pathways, mitigating damage related to abnormal hypoxic responses, which may be the main mechanism through which it protects the kidneys.

## 1. Introduction

Diabetic kidney disease (DKD) is a progressive renal condition caused by chronic diabetes mellitus. It is characterized by persistent albuminuria and a decline in renal function, making it a leading global cause of end-stage renal disease [1]. The complex pathophysiological mechanisms of DKD present significant challenges for both its prevention and management [2]. Recent studies suggest that chronic hypoxia, particularly within the renal tubular interstitium, plays a key role in the progression of all chronic kidney diseases. This phenomenon is especially pronounced in DKD [3].

The kidneys are highly susceptible to both acute and chronic hypoxic damage, and chronic hypoxia is a notable feature of DKD [4]. This condition arises from a mismatch between oxygen delivery and oxygen demand. The high filtration state and increased glucose reabsorption in DKD significantly raise the kidney's oxygen demand [5]. Additionally, under hyperglycemic conditions, energy metabolism shifts from glucose oxidation to free fatty acid oxidation, which reduces the efficiency of oxygen utilization [6, 7]. Chronic inflammation–induced fibrosis and reduced perirenal capillaries further impair oxygen delivery and diffusion [8]. Moreover, hypoxic conditions can also induce inflammation and fibrosis, further exacerbating renal hypoxia [9].

Chronic hypoxia is primarily regulated by HIF-1*α*, a key mediator of the hypoxic response. HIF-1*α* facilitates kidney adaptation to hypoxic conditions by inducing angiogenesis and regulating erythropoietin production, both of which play a crucial role in the pathogenesis and progression of DKD [10]. However, persistent and excessive hypoxic responses can have negative effects. The angiogenic actions of HIF-1*α* are often accompanied by the production of proinflammatory cytokines and the activation of profibrotic mechanisms [11]. Reports indicate that hypoxia activates profibrotic signaling pathways, including TGF-*β*/SMAD3, NF-*κ*B, and TNF-*α*, in an HIF-1*α*-dependent manner. HIF-1*α* knockout mice have been shown to inhibit these pathways, thereby alleviating renal tubular interstitial inflammation and fibrosis [12, 13]. Therefore, targeting the regulation of hypoxic responses holds significant potential for treating DKD, and the development of relevant medications is urgently needed.

Traditional Chinese medicine (TCM) is based on a holistic theory that emphasizes the relationship between human and nature, and it has a history of using natural herbs to combat diseases for thousands of years [14]. TCM has accumulated extensive experience over the course of history in treating DKD, and it has become a viable alternative therapy for the condition. The QGSJS decoction combines the classic TCM formulas Dang-Gui-Bu-Xue-Tang (DGBXT) and Sheng-Jiang-San (SJS), which include Huangqi (*Astragalus membranaceus* (Fisch.) Bunge), Danggui (*Angelica sinensis* (Oliv.) Diels), Jiangcan (*Bombyx batryticatus*), Jianghuang (*Curcuma longa* L.), Chantui (Cicadae periostracum), and Dahuang (*Rheum palmatum* L.). In TCM, DGBXT, which is composed of Huangqi and Danggui, is a classic formula for tonifying Qi and blood. It originates from Li Dongyuan's “Neiwaishangbianhuolun” of the Yuan Dynasty. Reports indicate that DGBXT possesses antihypoxic and immune-regulatory effects, reduces urinary protein, and is used in the treatment of DKD [15, 16]. SJS, composed of Jiangcan, Chantui, Jianghuang, and Dahuang, is a classic formula for dispelling wind and detoxifying. It originates from Yang Lisan's “Shanghanwenyitiaobian” of the Qing Dynasty. It is widely used to treat infectious and inflammatory diseases and has been reported to have a beneficial effect on renal function [17]. QGSJS decoction has been used in the treatment of DKD for over a decade. This treatment reflects a method of tonifying Qi, invigorating blood, and dispelling wind, which can enhance the circulation of Qi and blood, may have potential antihypoxic effects, and warrants further investigation.

**Figure 1:**
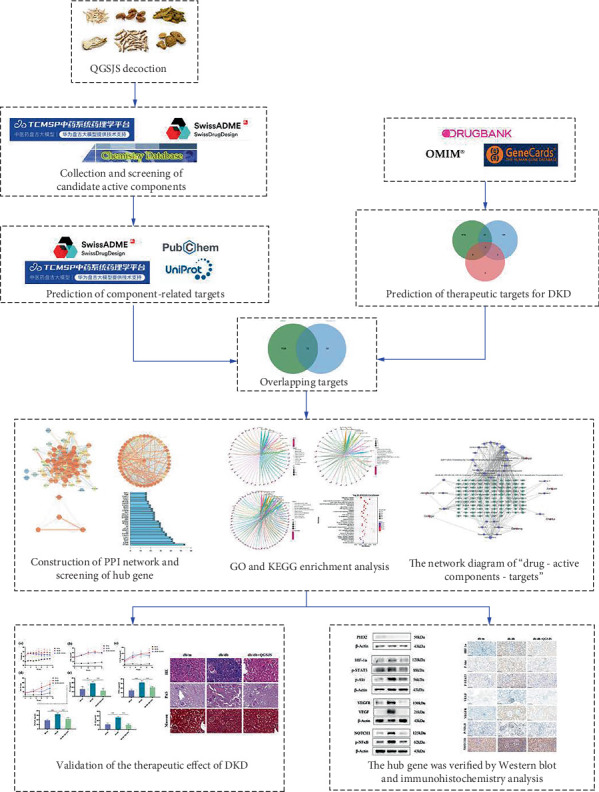
The flowchart of network pharmacology–based strategy for deciphering the mechanisms of QGSJS decoction acting on DKD.

As modern medicine's understanding of complex diseases has advanced, research into drug mechanisms has shifted from the traditional “single-target, single-compound” model to the “multitarget, multicompound” model, which emphasizes the systemic regulation of multiple targets [18]. Network pharmacology, as a novel paradigm for elucidating compound-gene-disease system interactions, facilitates the exploration of TCM formula mechanisms, providing new insights and directions for their study [19]. Therefore, this study initially investigated whether the mechanism of QGSJS decoction involves the regulation of hypoxic responses through network pharmacology. Subsequently, we evaluated its efficacy in improving urinary protein levels and renal function through animal experiments and validated its mechanism using molecular biology techniques. Our research procedure is shown in Figure 1.

## 2. Materials and Methods

### 2.1. Collection and Screening of Candidate Active Components

Major candidate chemical components were obtained from the Traditional Chinese Medicine Systems Pharmacology (TCMSP) database (https://old.tcmsp-e.com/tcmsp.php) and the Chemistry Database (http://www.organchem.csdb.cn) [20, 21]. The screening criteria for the TCMSP database were set as oral bioavailability (OB) ≥ 30% and drug-likeness (DL) ≥ 0.18 [6]. Additional components of Jiangcan and Chantui were identified using the Chemistry Database, with their structures retrieved and downloaded from PubChem (https://pubchem.ncbi.nlm.nih.gov). The SwissADME tool (http://www.swissadme.ch/index.php) was employed to screen the chemical components corresponding to these structures, using criteria for high OB and DL that met at least two of the Lipinski, Ghose, Veber, Egan, or Muegge standards [22].

### 2.2. Prediction of Component-Related Targets

After retrieving the structural formulas of the active components from the PubChem database, SwissTargetPrediction (https://www.swisstargetprediction.ch/) was employed to predict the target genes for these components in the QGSJS decoction, specifying “human” as the species. Targets with a prediction probability of ≥ 0.5 were included. Simultaneously, the TCMSP database was utilized to identify and predict the relevant targets for the active components in the QGSJS decoction. The UniProtKB search page of the UniProt database (https://www.uniprot.org/) was employed to standardize the naming of all identified target genes and proteins, limiting the search to the human species.

### 2.3. Prediction of Therapeutic Targets for DKD

DKD-related targets were obtained from three databases: GeneCards (https://www.genecards.org/), which provides comprehensive and authoritative annotations of human genes [23]; OMIM (Online Mendelian Inheritance in Man) (https://www.omim.org/, updated April 22, 2024), a continually updated resource on human Mendelian genetic disorders, emphasizing the relationship between gene variations and phenotypic traits [24]; and DrugBank (https://go.drugbank.com/), a database from the University of Alberta that integrates detailed drug data and comprehensive target information, validated through experimental data and reliable bioinformatics and cheminformatics sources [25]. Using “diabetic kidney disease” as a search term, targets retrieved from the OMIM and DrugBank databases were merged with those from the GeneCards database, and duplicates were removed. The Venn online tool (https://bioinformatics.psb.ugent.be/webtools/venn/) was employed to identify overlapping targets. Cytoscape (Version 3.7.0) was used to construct a network diagram of “drug-active components-targets” for result visualization.

### 2.4. Protein–Protein Interaction (PPI) Data and Network Construction

The STRING database (https://cn.string-db.org/) was used to construct a PPI network. The overlapping targets of the QGSJS decoction and DKD were imported into the STRING database [26], with the biological species set to “*Homo sapiens*” for analysis. Nodes without interactions in the retrieval results were excluded, and the minimum interaction confidence threshold was set to “medium confidence” (≥ 0.4). The results were exported as a TSV file and visualized using Cytoscape (Version 3.7.0). The TSV file obtained from the online analysis was imported into R software. PPIs in the network were quantified using code scripts, and hub genes were ranked based on node degree. Among the top 30 ranked hub genes, those directly related to DKD and of significant interest for DKD treatment were selected. Their functional networks were further analyzed to identify core targets.

### 2.5. Gene Ontology (GO) and Kyoto Encyclopedia of Genes and Genomes (KEGG) Enrichment Analysis

GO is a database established by the Gene Ontology Consortium that uses a dynamically updated set of controlled vocabularies to describe the roles of genes and proteins in cellular processes. This database provides a comprehensive description of the attributes of genes and gene products within biological organisms. The GO database comprises three major categories: cellular component (CC), molecular function (MF), and biological process (BP). These categories describe the MFs of gene products, their cellular locations, and the BPs they participate in. The KEGG database systematically analyzes the metabolic pathways and functions of gene products in cells. It facilitates the study of gene expression information within an integrated network. OmicShare tools (https://www.omicshare.com/tools/) were used to conduct GO and KEGG enrichment analysis on common target genes to identify relevant functions and pathways with a significance level of *p* < 0.05.

### 2.6. Animals

Eight-week-old SPF-grade male spontaneous Type 2 diabetic db/db mice and their wild-type littermates (db/m) were purchased from Changzhou Cavens Experimental Animals Co. Ltd. [Animal License Number SCXK (Su) 2016-0010]. The animals were housed under conditions with a temperature range of 20°C–26°C, a daily temperature variation of ≤ 3°C, relative humidity of 40%–70%, and a 12-h light/dark cycle.

### 2.7. Drugs and Reagents

The Chinese herbal medicines used in the QGSJS decoction were uniformly purchased and identified by the Herbal Pharmacy of Dongzhimen Hospital, Beijing University of Chinese Medicine. The herbs were combined, boiled in 500 mL of distilled water for 30 min, filtered, and collected. This process was repeated twice. The two decoctions were then mixed and concentrated to a final concentration of 1092 mg/mL. This decoction is a hospital preparation. The dosage was determined based on commonly used clinical doses, calculated according to body weight. For mice, the dosage was calculated using methods described in the literature [27], and it was 9.1 times that for humans. The concentrations of each component are as follows: Huangqi 436.8 mg/mL, Danggui 145.6 mg/mL, Jiangcan 145.6 mg/mL, Jianghuang 145.6 mg/mL, Chantui 145.6 mg/mL, and Dahuang 72.8 mg/mL.

Antibodies, including p-Akt, p-STAT3, and STAT3, were purchased from Abcam (ab81283, ab76315, and ab68153). Antibodies, including p-NF-*κ*B and VEGF, were purchased from Santa Cruz (sc-136548, sc-53462), and the VEGFR antibody was obtained from Bioss (Bsm-52338R). Antibodies, including HIF-1*α* and PHD2, were obtained from ABclonal (A11945, A14557), and the NOTCH1 antibody was purchased from Proteintech (20687-1-AP). Measurement kits for glycated serum albumin (GSP), triglycerides (TG), total cholesterol (TC), creatinine (Cr), and blood urea nitrogen (BUN) were obtained from Nanjing Jiancheng Bioengineering Institute (A037-2-1, A110-1-1, A111-1-1, C011-2-1, and C013-2-1). The microalbuminuria assay kit was obtained from Shanghai Enzyme-Linked Biotechnology Co. Ltd. (Catalog Number ML063626-2). Enzyme-linked immunosorbent assay (ELISA) kits for transforming growth factor *β*1 (TGF-*β*1), mouse interleukin-1*β* (IL-1*β*), mouse interleukin-6 (IL-6), mouse tumor necrosis factor *α* (TNF-*α*), and mouse insulin (INS) were obtained from Elabscience (E-EL-0162, E-MSEL-M0003, E-MSEL-M0001, E-MSEL-M0002, and E-EL-M1382c).

### 2.8. Ingredient Analysis of QGSJS Decoction

The HPLC-MS system was used to identify the target compounds responsible for their therapeutic effects on DKD. Huangqi, Danggui, Jiangcan, Jianghuang, Chantui, and Dahuang were made into a test solution by mixing their lyophilized powders at a ratio of 5:2:2:2:2:2. A 1-*μ*L aliquot of both solutions was analyzed by an Agilent UPLC 1260 Infinity II-Ultivo-QQQ system. The UPLC, equipped with a ZORBAX Eclipse Plus C18 column (1.8 *μ*m, 50 × 2.1 mm), was set at 26°C. Acetonitrile (A) and 0.1% formic acid aqueous solution (B) were used for gradient elution (0–3 min, 18% ⟶ 60% A; 3–7 min, 60% ⟶ 95% A; 7–10 min, 95% A) at 0.2 mL/min. Mass spectrometry electrospray ionization source (ESI), multireaction mode (MRM) detection, the drying gas was at 300°C (10 L·min^−1^), the atomizing gas pressure was 20 psi, and the sheath gas was at 250°C (10 L·min^−1^). Peak orders and retention times of 11 components in both solutions were compared under these chromatographic conditions.

### 2.9. Grouping and Intervention

After a 1-week acclimatization period, the mice were randomly divided into three groups: db/m, db/db, and db/db + QGSJS (*n* = 6 per group). The db/db + QGSJS group was administered QGSJS decoction via gavage at a dose of 0.01 mL/g body weight, while the db/m and db/db groups were given an equivalent volume of distilled water, orally once daily for 9 consecutive weeks. During the experiment, the mice were fed an SPF-grade standard diet and had ad libitum access to water.

### 2.10. Specimen Collection

Body weight was measured weekly, and fasting blood glucose (FBG) from the tail vein was measured every 2 weeks after a 6-h fast. Every 3 weeks, urine samples were collected over 6 h using metabolic cages, beginning after the gastric gavage with distilled water, with fasting and water restriction observed. After completing the drug intervention, the mice were fasted for 12 h, anesthetized with an intraperitoneal injection of 3% sodium pentobarbital (1.6 mL/kg), and then sacrificed. Blood samples were allowed to stand at room temperature for 1 h and then centrifuged at 3000 rpm for 15 min at 4°C before subsequent analysis. The kidneys were isolated and sectioned longitudinally. One half of each kidney was fixed in 4% paraformaldehyde, while the other half was stored in a −80°C freezer for subsequent analysis.

### 2.11. Urine Protein

Urine samples from each group of mice were collected over 6 h and then centrifuged at 4°C and 3000 rpm for 15 min. Urine volume was measured, and the supernatant was analyzed for urinary Cr and microalbumin levels. The urinary microalbumin-to-creatinine ratio (ACR) and the 6-h urinary microalbumin (6hUTP) levels were calculated from these measurements.

### 2.12. HE, PAS, and Masson Staining

After fixation, tissues were dehydrated in graded ethanol, cleared, infiltrated with paraffin, embedded, and sectioned at a thickness of 0.4 *μ*m. Sections were stained with hematoxylin and eosin (HE), periodic acid-Schiff (PAS), and Masson's trichrome stains and then examined for renal pathological changes using an optical microscope.

### 2.13. Biochemical Assays

Serum levels of Cr, BUN, glycated serum protein (GSP), TC, and TG were measured using specific assay kits, following the manufacturer's instructions.

### 2.14. ELISA

ELISA was utilized to quantify INS, TGF-*β*1, IL-1*β*, IL-6, and TNF-*α* by the manufacturer's instructions. The insulin resistance index (HOMA-IR) and the insulin sensitivity index (HOMA-IS) were calculated from FBG and INS measurements using the formulas HOMA-IR = FBG (mmol/L) × INS (mIU/L)/22.5 and HOMA-IS = 1/HOMA-IR [28].

### 2.15. Immunohistochemistry

Immunohistochemical analysis of renal HIF-1*α*, VEGF, VEGFR, NOTCH1, p-STAT3, p-NF-*κ*B, and p-Akt was performed as previously described [29]. Tissue staining was examined under a microscope. Five random fields per slide were photographed, each containing at least one glomerulus. Image analysis was conducted using ImageJ software to measure the average optical density.

### 2.16. Western Blot

Western blot was done as previously described [30]. Protein band intensities were quantified using ImageJ software.

### 2.17. Statistical Analysis

Experimental data were analyzed with SPSS 26.0 software. Data distribution normality was assessed, followed by tests for homogeneity of variances. Group comparisons were conducted using one-way analysis of variance (ANOVA). When variances were homogeneous, pairwise comparisons were made using the LSD test; otherwise, the Dunnett T3 test was used. A significance level of *p* < 0.05 was considered statistically significant, and *p* < 0.01 was regarded as indicating highly significant differences.

## 3. Result

### 3.1. Active Components of QGSJS Decoction

A total of 41 components were retrieved from the TCMSP database: 20 from Huangqi, 2 from Danggui, 16 from Dahuang, and 3 from Jianghuang (Table 1). Supplementary data on active components were also obtained from a chemical database (https://organchem.csdb.cn). After screening with SwissADME, 18 active components were identified: 4 from Danggui, 3 from Jianghuang, 2 from Chantui, and 9 from Jiangcan (Table 2). After removing the duplicate components, a total of 57 components were obtained.

### 3.2. Prediction of DKD-Associated Targets

**Figure 2:**
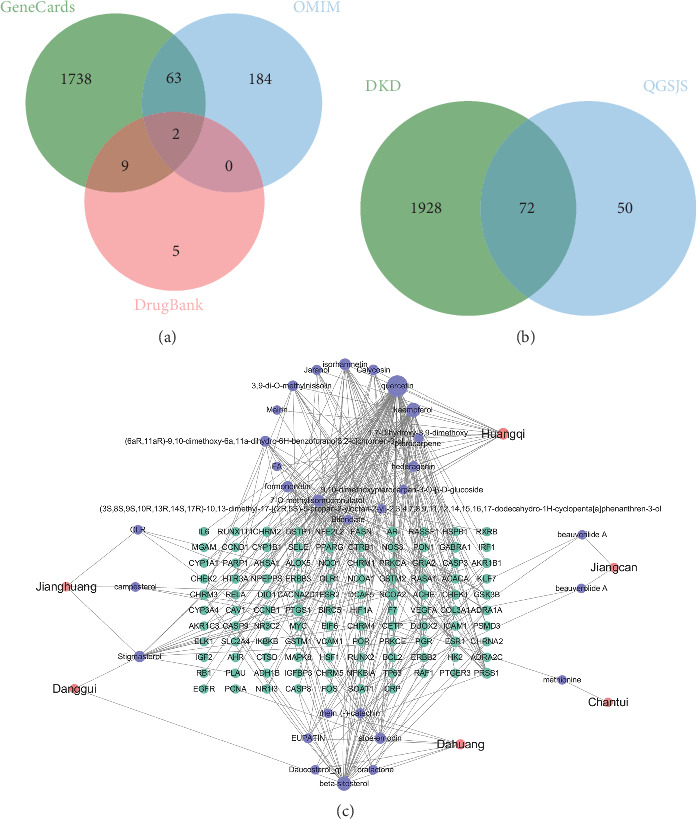
Prediction of drug-related targets of QGSJS decoction in DKD. (a) The Venn diagram of targets in GeneCards, OMIM, and DrugBank. (b) The Venn diagram of targets both in DKD targets and QGSJS decoction's targets. (c) A network diagram of “drug-active components-targets” of the QGSJS decoction treating DKD. Note: The size of the nodes is proportional to the degree of the targets in the network.

Among the 2057 targets associated with the 57 components, 122 unique target genes were identified following filtering and deduplication. The GeneCards database identified 13,409 genes linked to DKD. Applying a filtering criterion set at twice the median relevance score (scores greater than 15), 1811 genes were selected. Searches of the OMIM and DrugBank databases identified 249 and 16 genes, respectively (Figure 2a). By combining and deduplicating these results, a total of 2000 DKD-related genes were identified. Using the Venn online tool, 72 overlapping targets between the QGSJS decoction and DKD were identified (Figure 2b). Drug, active components, and targets form an interplay network for DKD treatment (Figure 2c).

### 3.3. Construction of the PPI Network

**Figure 3:**
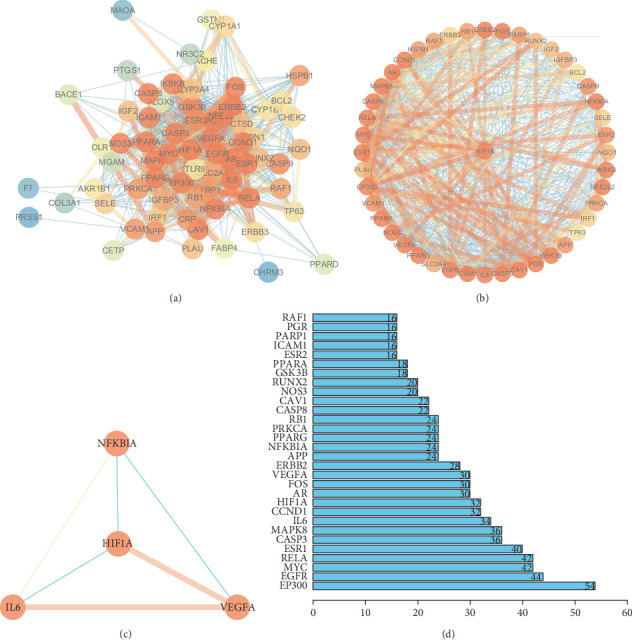
(a) PPI network of all QGSJS decoction anti-DKD targets. (b) PPI network with HIF1A as a hub gene. (c) HIF1A, NFKB1A, IL6, and VEGFA interact with each other, which may be a potential target of QGSJS decoction anti-DKD. (d) Hub genes and their correlation with anti-DKD. Note: In the PPI network, nodes represent genes, targets, proteins, or molecules, while edges represent interactions among these entities.

The constructed PPI network identified 71 target proteins associated with QGSJS decoction for the treatment of DKD. Among the top 10 hub genes with the highest connectivity, HIF1A, a key regulator of hypoxic responses, was included (Figure 3a,d). By focusing on HIF1A as the hub gene for protein interaction analysis, we found that IL6, NFKBIA, HIF1A, and VEGFA are closely related. These genes are involved in the inflammatory-hypoxic-abnormal angiogenesis pathology [11], which aligns with the pathological mechanisms of DKD and may represent the primary targets of QGSJS decoction for treating DKD (Figure 3b,c).

### 3.4. GO and KEGG Enrichment Analysis

**Figure 4:**
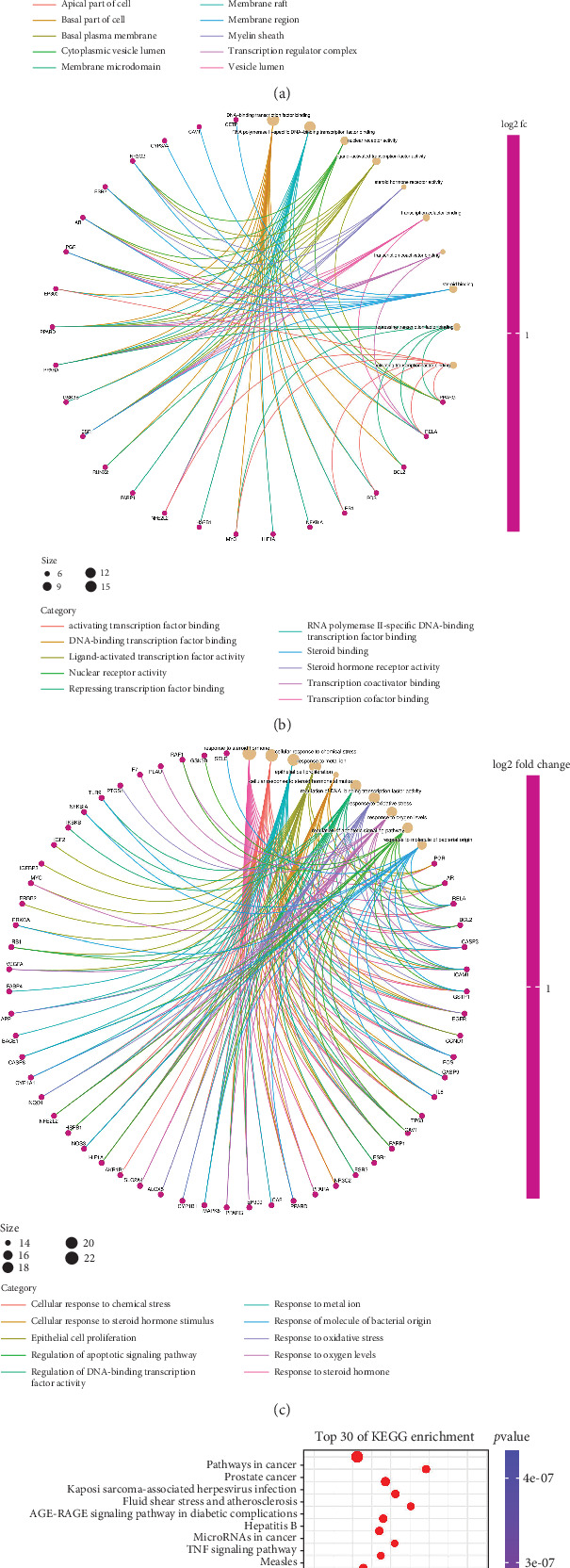
Enrichment analysis of biological processes and pathways. (a–c) GO enrichment analysis for potential targets of QGSJS decoction in anti-DKD. These sections, respectively, correspond to the analyses of cellular components (CCs), molecular functions (MFs), and biological processes (BPs). (d) KEGG pathway enrichment analysis for potential targets of QGSJS decoction in anti-DKD. (*q* value refers to −log10 *p* value).

GO analysis was performed to further explore the biological functions of the 72 targets of QGSJS decoction. Results reveal that CCs associated with HIF-1*α* are primary components of the transcription regulator complex. Key BPs involving HIF-1*α* include cellular responses to chemical stress, metal ions, epithelial cell proliferation, and oxidative stress. The main MFs associated with HIF-1*α* include DNA-binding transcription factor binding, RNA polymerase II-specific binding, and additional DNA-binding transcription factor interactions (Figures 4a, 4b, and 4c).

Besides GO analysis, KEGG enrichment analysis was also performed, selecting the top 30 pathways based on count values and *p* values (Figure 4d). Key pathways related to DKD include the HIF-1 signaling pathway, AGE-RAGE signaling pathway, apoptosis, TNF-*α* signaling pathway, and INS resistance [31]. In summary, the therapeutic effect of QGSJS decoction on DKD may be attributed to its modulation of the body's response to hypoxia, oxidative stress, and inflammation. Regulation of the hypoxic response may be the primary mechanism through which QGSJS decoction treats DKD, with additional effects on inflammation and oxidative stress.

### 3.5. Components of QGSJS Decoction by HPLC-MS

**Figure 5:**
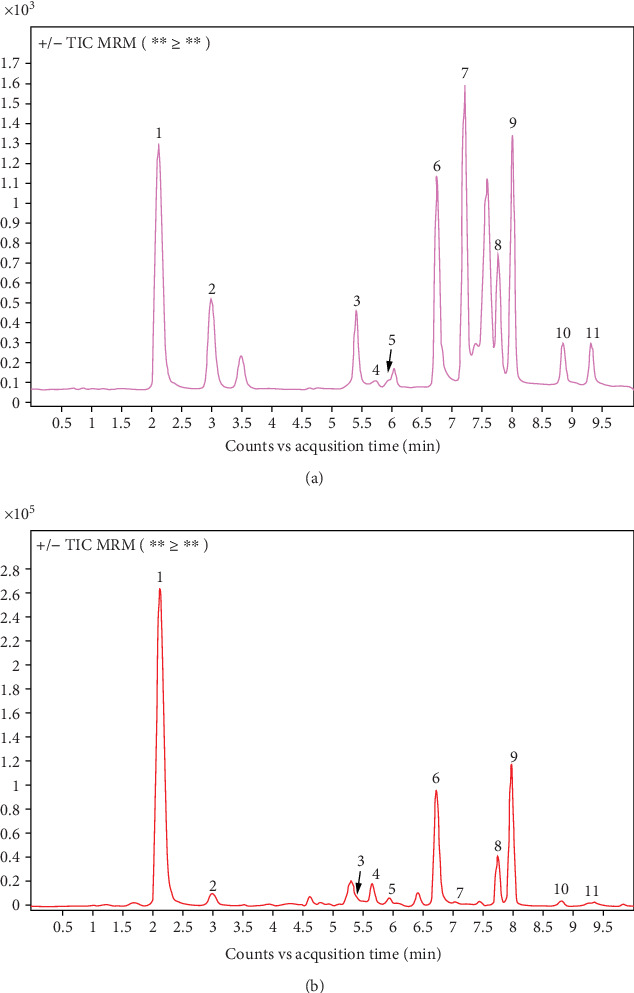
Total ion flow in positive and negative ion mode of QGSJS decoction and mixed control solution. (a) Mixed control solution. (b) QGSJS decoction. Note: 1—calycosin glucoside; 2—ferulic acid; 3—quercetin; 4—astragaloside IV; 5—kaempferol; 6—emodin; 7—curcumin; 8—chrysophanol; 9—ligustilide; 10—chrysophanol; 11—chrysophanol methyl ether.

By analyzing the retention times, precise molecular weights, and fragmentation ion data of the compounds using HPLC-MS, 11 chemical components were identified in the QGSJS decoction (Figure 5). Most of the identified components were consistent with those obtained through previous screenings.

### 3.6. Body Weight and Blood Glucose

**Figure 6:**
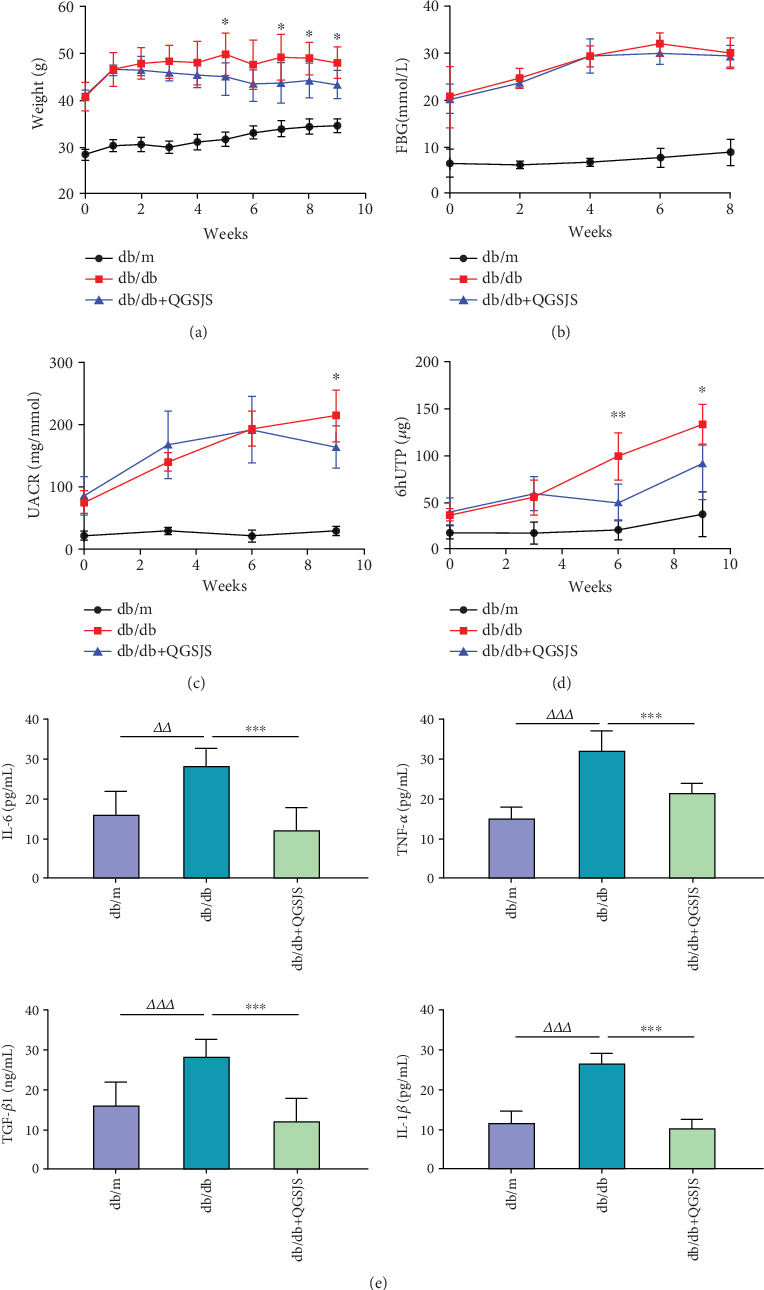
The effects on the main therapeutic indexes and cytokines. (a–d) The changes in weight, FBG, UACR, and 6hUTP throughout. (e) ELISA was used to measure serum cytokine levels. Statistical comparisons (*n* = 6): db/db versus db/db + QGSJS (⁣^∗^*p* < 0.05, ⁣^∗∗^*p* < 0.01, ⁣^∗∗∗^*p* < 0.001); db/m versus db/db (^△△^*p* < 0.01, ^△△△^*p* < 0.001).

Figure 6a demonstrates that the body weight of the db/db group was consistently higher than that of the db/m group throughout the experiment (*p* < 0.001), with an upward trend during the first 5 weeks. Weight gain in the db/db + QGSJS group was attenuated, with body weight lower than that of the db/db group at Weeks 5, 7, 8, and 9 (*p* < 0.05). Blood glucose levels were significantly elevated in the db/db group compared to the db/m group (*p* < 0.001), confirming the diabetic status of db/db mice (Figure 6b). No significant difference in FBG levels was observed between the db/db + QGSJS and db/db groups. The blood glucose levels of db/db mice often showed elevated values during the detection process, which may have affected the statistical results. GSP, reflecting the average blood glucose level over the past 2–3 weeks, was significantly lower in the db/db + QGSJS group compared to the db/db group (Table 3). Combined with the FBG results, this suggests that QGSJS decoction may primarily affect postprandial glucose levels and glucose fluctuations. HOMA-IR and HOMA-IS were calculated using FBG and INS levels from Week 9. The db/db group showed increased HOMA-IR and decreased HOMA-IS compared to the db/m group. However, the db/db + QGSJS group partially reversed these changes, indicating improved INS resistance.

### 3.7. Proteinuria

Figure 6c indicates that ACR in the db/m group did not show a significant increase. In contrast, ACR in the db/db group was significantly elevated each week compared to the db/m group (*p* < 0.001) and showed a rising trend over time, indicating kidney damage in db/db mice. By Week 9, ACR in the db/db + QGSJS group was significantly lower than that in the db/db group. Six hUTP levels in the db/m group remained relatively low. The db/db group exhibited an increasing trend in 6 hUTP over time compared to the db/m group, while the db/db + QGSJS group significantly delayed this increase (Figure 6d).

### 3.8. Biochemical Indicators

Table 3 shows that levels of Cr, BUN, TG, and TC were significantly elevated in the db/db group compared to the db/m group. The db/db + QGSJS group significantly reduced levels of Cr, BUN, and TC compared to the db/db group, indicating an improvement in glomerular filtration rate and a beneficial effect on lipid metabolism.

### 3.9. HE, PAS, and Masson Staining

**Figure 7:**
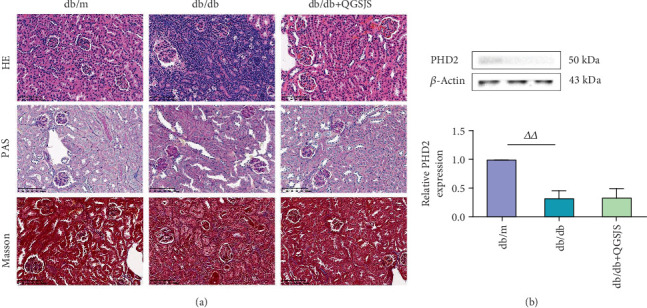
(a) Light microscopic observation of kidney tissue (magnification: 200×). (b) Western blot analysis of PHD2 expression. The groups from left to right are db/m, db/db, and db/db + QGSJS. Statistical comparisons (*n* = 6): db/m versus db/db (^△△^*p* < 0.01).

In Figure 7a, HE staining revealed glomerular hypertrophy, severe capillary loop expansion, congestion, and narrowing of the glomerular capsules in the db/db group, alongside vacuolar degeneration in renal tubular epithelial cells and severe congestion, edema, and extensive inflammatory cell infiltration in the renal interstitium. The extent of these changes was milder in the db/db + QGSJS group. PAS and Masson staining revealed mesangial cell proliferation, mesangial matrix expansion, and renal interstitial fibrosis in the db/db group. These changes were less severe in the db/db + QGSJS group.

### 3.10. Inflammatory Factors

Figure 6e shows that, compared to the db/m group, the db/db group exhibited significantly higher levels of TNF-*α*, IL-6, IL-1*β*, and TGF-*β*1. In contrast, the db/db + QGSJS group showed significantly lower levels of these inflammatory factors compared to the db/db group, suggesting an improved immune regulatory effect.

### 3.11. Western Blot Analysis of Kidney Expression of HIF-1*α*, p-Akt, VEGF, VEGFR, p-STAT3, p-NF-*κ*B, and NOTCH1

**Figure 8:**
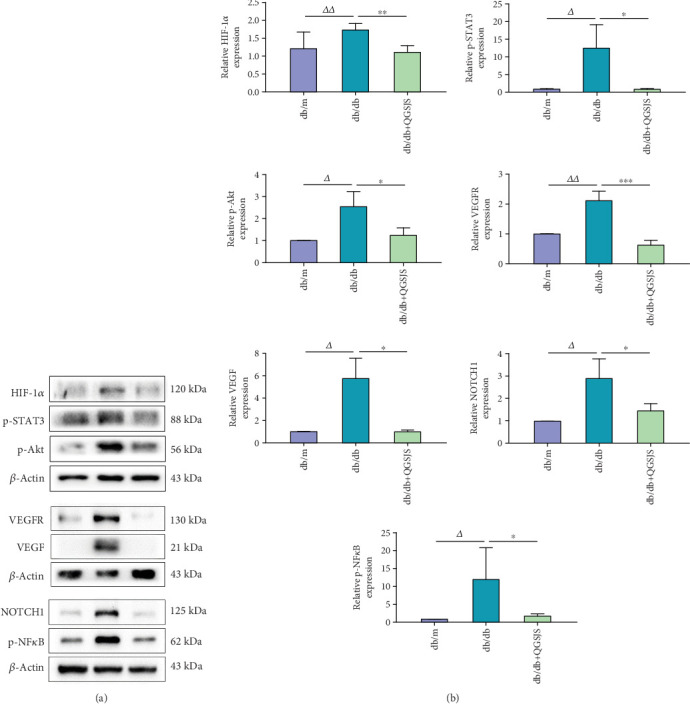
(a) Western blot analysis of the expression levels of HIF-1*α*, p-STAT3, p-Akt, VEGF, VEGFR, NOTCH1, and p-NF*κ*B. The groups from left to right are db/m, db/db, and db/db + QGSJS. (b) Statistical comparisons (*n* = 6): db/db versus db/db + QGSJS (⁣^∗^*p* < 0.05, ⁣^∗∗^*p* < 0.01, ⁣^∗∗∗^*p* < 0.001); db/m versus db/db (^△^*p* < 0.05, ^△△^*p* < 0.01).

Figure 8 illustrates that, compared to the db/m group, the db/db group had significantly higher levels of HIF-1*α*, p-STAT3, p-Akt, VEGF, VEGFR, p-NF-*κ*B, and NOTCH1, whereas PHD2 levels were markedly reduced (Figure 7b). In comparison, the db/db + QGSJS group exhibited lower levels of these proteins relative to the db/db group, with no significant change in PHD2 levels.

### 3.12. Immunohistochemical Analysis of Kidney Expression of HIF-1*α*, p-Akt, VEGF, VEGFR, p-STAT3, p-NF-*κ*B, and NOTCH1

**Figure 9:**
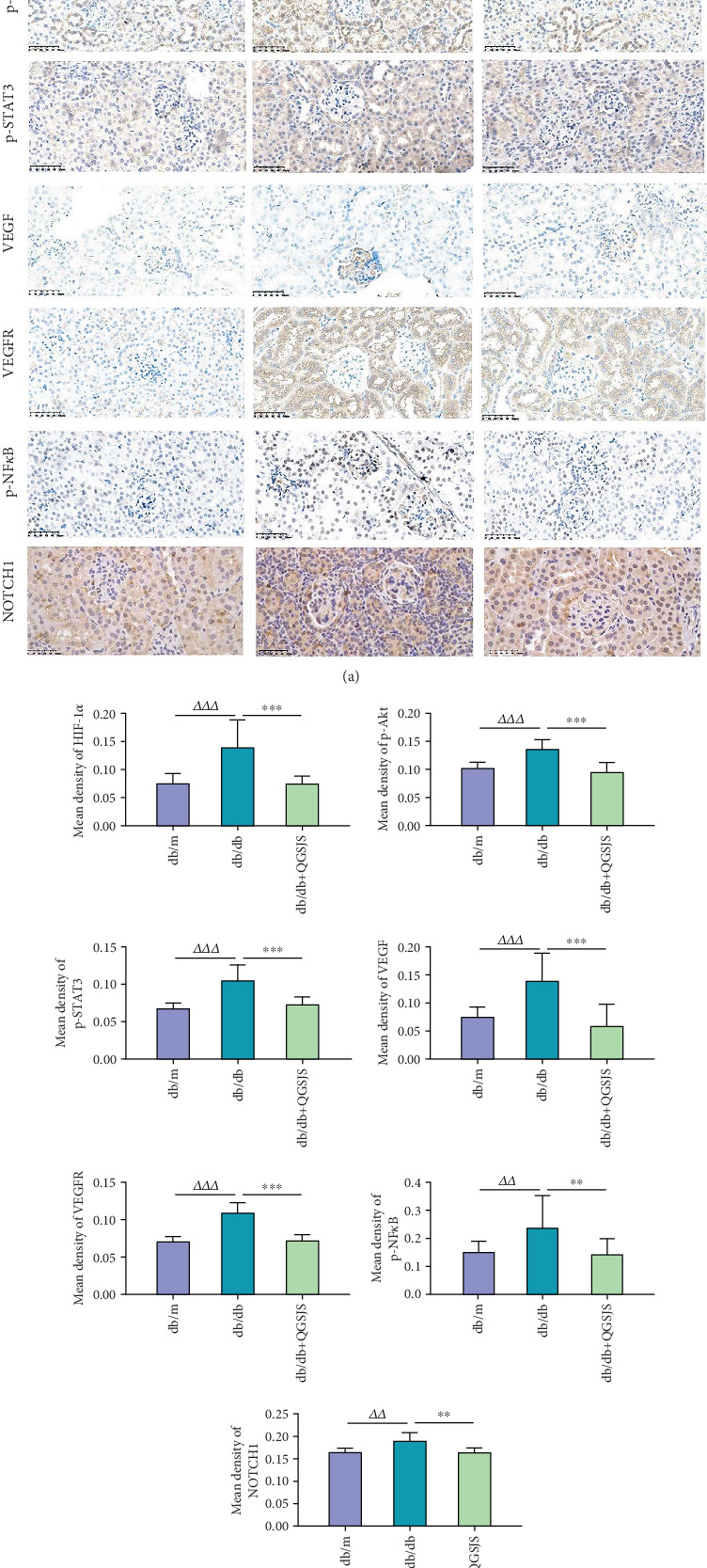
Immunohistochemical observation of the expression status of HIF-1*α*, p-STAT3, p-Akt, VEGF, VEGFR, p-NF*κ*B, and NOTCH1. (a) Representative images of immunohistochemistry. (b) Statistical results. Statistical comparisons (*n* = 3): db/db versus db/db + QGSJS (⁣^∗∗^*p* < 0.01, ⁣^∗∗∗^*p* < 0.001); db/m versus db/db (^△△^*p* < 0.01, ^△△△^*p* < 0.001).

Immunohistochemical staining of kidney tissue was performed and observed under a microscope at 400× magnification, with positive staining appearing as brownish–yellow areas.  Figure 9 displays the distribution of positive staining areas and the average optical density of protein expression for HIF-1*α*, p-Akt, VEGF, VEGFR, p-STAT3, p-NF-*κ*B, and NOTCH1 in kidney tissues from each mouse group. These markers were present in both glomeruli and renal tubules: HIF-1*α*, p-Akt, VEGFR, p-STAT3, and NOTCH1 were primarily localized in the renal tubules; VEGF was mainly found in the glomeruli; and p-NF-*κ*B was predominantly localized in the cell nuclei. The db/db group exhibited significantly higher levels of HIF-1*α*, p-Akt, VEGF, VEGFR, p-STAT3, p-NF-*κ*B, and NOTCH1 compared to the db/m group. In contrast, the db/db + QGSJS group showed reduced levels of these proteins relative to the db/db group.

## 4. Discussion

DKD is characterized by a rapid decline in renal function and is associated with chronic hypoxia and an excessive hypoxic response, which play a pivotal role in the exacerbation of renal inflammation and fibrosis [2]. Recently, TCM has gained international recognition for its effectiveness and has made significant contributions to DKD treatment [14]. According to TCM theory, DKD is referred to as “edema” and “kidney elimination,” with its pathological mechanisms categorized as qi deficiency, blood stasis, heat toxicity, and wind pathogen. QGSJS decoction contains SGBXT and SJS, both of which have been used for over 100 years, and their efficacy is consistent with the pathological mechanisms of DKD as outlined in TCM theory.

The results of this study indicate that QGSJS decoction improved urinary protein, renal function, body weight, and glucose–lipid metabolism in db/db mice, demonstrating its therapeutic potential for DKD. Modern pharmacology has reported that Huangqi, Danggui, Jianghuang, and Dahuang regulate hypoxic responses and immune inflammation [15, 32, 33]. TCM emphasizes using herbal formulas rather than individual herbs for disease treatment. Chinese herbal formulas are characterized by flexible composition, diverse therapeutic methods, and complex chemical components. Focusing on only a few targets may hinder a systematic and comprehensive analysis [18]. This study employed network pharmacology to identify the primary components and targets of the QGSJS decoction, as well as to validate its mechanisms of action.

### 4.1. The Mechanism of Action of QGSJS Decoction Is Associated With the Regulation of Hypoxic Responses, as Indicated by Network Pharmacology Studies

We have screened 57 active ingredients and 72 potential targets for DKD using the method described above. The “drug component-target” network (Figure 3c) reveals that key components, including quercetin, kaempferol, and isorhamnetin, are likely essential for treating DKD. Quercetin has been reported to inhibit aerobic glycolysis via HIF-1*α* in mesangial cells, thereby mitigating renal damage caused by glucose fluctuations [34]. Kaempferol has been shown to enhance intracellular ATP levels under hypoxic conditions by inhibiting HIF-1*α* [35]. Isorhamnetin exhibits antioxidant, anti-inflammatory, and antiproliferative activities and can inhibit HIF-1*α* accumulation [36]. Considering the core components, the intrinsic mechanisms of QGSJS decoction in preventing and treating DKD likely involve combating hypoxic damage and exerting anti-inflammatory effects.

Analysis of the PPI network identified EP300, EGFR, MYC, RELA, ESR1, CASP3, MAPK8, IL6, CCND1, and HIF1A as the top 10 hub genes, respectively. Among these, HIF-1*α*, encoded by HIF1A, is a crucial protein in regulating the hypoxic response. Within the top 30 hub genes, HIF-1*α* has significant interactions with IL6, NFKBIA, and VEGFA, forming a distinct interaction network. IL-6 has been reported to induce the hypoxic response by activating the STAT3/HIF-1*α* pathway [37]. NFKBIA encodes I*κ*B, an inhibitor of the NF-*κ*B complex, which affects the synthesis and release of proinflammatory cytokines like TNF-*α*, IL-6, and IL-1*β*. It also connects immune inflammation with the hypoxic response through its regulatory effects on HIF-1*α* transcription. It has been confirmed that NF-*κ*B controls HIF-1*α* transcription in macrophages activated by LPS through chromatin immunoprecipitation (ChIP) [38]. The NF-*κ*B/HIF-1*α* signaling pathway exacerbates renal inflammation by mediating glycolysis and macrophage polarization [39]. A recent study demonstrated that the NF-*κ*B/HIF-1*α* signaling pathway promotes CXCL1 secretion from fibroblasts via glycolysis, inducing neutrophil infiltration [40]. VEGFA, a downstream target of HIF-1*α*, is highly expressed under hypoxic conditions [41]. These findings suggest that the potential targets of QGSJS decoction are related to the regulation of hypoxic responses and inflammation.

To further analyze whether the biological mechanisms of QGSJS decoction in the treatment of DKD are related to the hypoxic response, GO and KEGG enrichment analyses were conducted. These analyses predicted the top 10 MFs, BPs, and CCs involved in the 72 targets, as well as the top 30 signaling pathways. The meaningful results are shown above. The results of the GO enrichment analysis indicate that QGSJS decoction is involved in regulating BPs and MFs associated with HIF-1*α*. The KEGG enrichment analysis identified key pathways, such as HIF-1, NF-*κ*B, and TNF signaling pathways. The HIF-1 signaling pathway is a central pathway through which the body responds to hypoxic conditions and regulates the transcription of specific genes. It is closely associated with inflammation-related signaling pathways. HIF-1*α* is a key component of the hypoxic response, regulating hypoxia by modulating signaling pathways associated with inflammation, fibrosis, and angiogenesis. The activation of these pathways can also reflect hypoxic damage to a certain extent [42]. Based on the results of network pharmacology, this study selected and validated HIF-1*α* and several signaling pathways upstream and downstream of it.

### 4.2. Potential Regulation of HIF-1a Through Non–Oxygen-Dependent Pathways With QGSJS Decoction

HIF-1*α* is a key oxygen sensor and regulator of oxygen delivery and consumption. Under normoxic conditions, HIF-1*α* is degraded after hydroxylation by PHD, whereas its expression increases in the kidneys under hypoxic conditions [38]. HIF-1*α* can be activated through both oxygen-dependent and non–oxygen-dependent pathways, including PI3K/Akt, NF-*κ*B, and STAT3 signaling pathways [43]. The PI3K/Akt signaling pathway contributes to the hypoxic response by regulating proteins related to survival and apoptosis, thereby modulating HIF-1*α* expression. Akt is a central component of the PI3K/Akt pathway [44]. NF-*κ*B is central to nonspecific immune responses and serves as a crucial transcriptional activator of HIF-1*α*. Basal NF-*κ*B activity is essential for the accumulation of HIF-1*α* protein under hypoxic conditions [38]. STAT3 acts as a transcription factor and can be activated primarily by inflammatory factors such as IL-6. STAT3 is a downstream effector of proinflammatory mediators and is involved in the transcription and stabilization of HIF-1*α* [45, 46].

**Figure 10:**
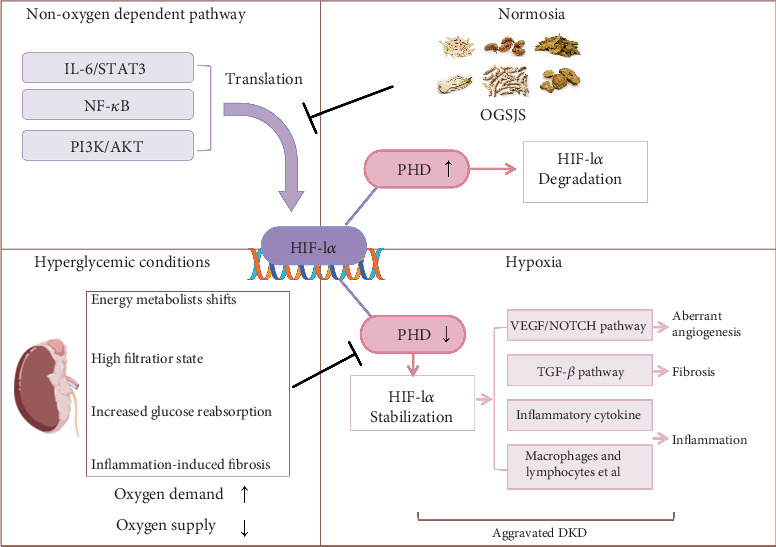
QGSJS decoction inhibits the expression of HIF-1*α* through non–oxygen-dependent pathways, thereby improving kidney damage caused by excessive hypoxic responses. In db/db mice, the hyperglycemic conditions lead to high renal perfusion, a shift in energy metabolism, increased glucose reabsorption, inflammation, and fibrosis, which results in a relatively hypoxic state. This prevents PHD from degrading HIF-1*α*, and the overexpression of HIF-1*α* activates mechanisms related to abnormal angiogenesis, fibrosis, and inflammation, thus promoting the progression of DKD. In addition to oxygen-dependent pathways, non–oxygen-dependent pathways such as IL6/STAT3, NF-*κ*B, and PI3K/AKT also regulate the transcription of HIF-1*α*. QGSJS decoction inhibits these pathways, thereby reducing HIF-1*α* expression and protecting the kidneys.

The results show that in the db/db group, HIF-1*α* expression was elevated in kidney tissue, while PHD expression was reduced. This suggests a hypoxic state in the kidneys and the activation of the hypoxic response. In the db/db + QGSJS group, HIF-1*α* expression was decreased, while PHD expression remained unchanged. This suggests that the QGSJS decoction inhibited the hypoxic response. However, hypoxia persisted in the kidneys, indicating that the QGSJS decoction may have had a limited effect on HIF-1*α* through oxygen-dependent pathways. Based on the network pharmacology results, we further analyzed its primary upstream pathways. The findings show that QGSJS decoction intervention led to a downregulation of p-Akt, p-NF-*κ*B, and p-STAT3 expression. This indicates that QGSJS decoction may inhibit HIF-1*α* by suppressing the activation of these non–oxygen-dependent signaling pathways (Figure 10).

### 4.3. QGSJS Decoction Alleviates Kidney Damage Induced by Persistent Activation of HIF-1*α*

HIF-1*α* is a mediator of the hypoxic response, which enables the body to adapt to hypoxia. However, it also has detrimental effects, which contribute to hypoxic damage. Angiogenesis represents a pivotal consequence of the hypoxic response, orchestrated by HIF-1*α*, with VEGF serving as a principal angiogenic factor exhibiting a pronounced sensitivity to hypoxic stimuli. In hypoxic renal tubules of DKD, HIF-1*α* rapidly accumulates and promotes the expression of VEGF and its receptor VEGFR [47]. The NOTCH1 signaling pathway is a highly conserved intercellular pathway that plays a pivotal role in angiogenesis. It interacts with both HIF-1*α* and VEGF and is sensitive to hypoxia [48, 49]. These pathways serve to compensate for hypoxic conditions by enhancing endothelial cell proliferation, promoting new blood vessel formation, and increasing vascular permeability. However, incomplete development of the blood vessels can disrupt the normal physiological structure of the kidneys, worsening proteinuria and glomerulosclerosis. The results of this study demonstrate that QGSJS decoction downregulates the expression of VEGF, VEGFR, and NOTCH1 in the kidneys of db/db mice, thereby improving urinary protein levels and kidney function. This suggests that QGSJS decoction may inhibit abnormal angiogenesis under hypoxic conditions, thereby protecting the normal kidney structure.

Inflammation and fibrosis are closely linked to the hypoxic response. In the context of sustained hypoxia in DKD, renal tubular epithelial cells initiate inflammation and activate fibroblasts by recruiting inflammatory cells to the damaged stroma and secreting various fibrotic and inflammatory factors, such as TNF-*α*, IL-6, IL-1*β*, and TGF-*β*1. This leads to epithelial-to-mesenchymal transition, tubular atrophy, and extracellular matrix accumulation. It has been reported that HIF-1*α* interacts with signaling pathways related to inflammation and fibrosis, such as TGF-*β*1/SMAD2/3, PI3K/AKT, NF-*κ*B, and TLR4, amplifying the inflammatory and fibrotic processes [50]. The results of this study demonstrate that QGSJS decoction markedly diminished serum levels of TNF-*α*, IL-6, IL-1*β*, and TGF-*β*1. Additionally, the pathological observations indicate that it improved renal inflammation and fibrosis. This effect may be achieved by the QGSJS decoction through the modulation of multiple signaling pathways, including the HIF-1*α*, STAT3, and NF-*κ*B pathways. Furthermore, activation of HIF-1*α* can result in the onset of inflammation in the pancreas and adipose tissue and may also influence signaling pathways associated with glucose and lipid metabolism, such as PPAR*α*/*γ*. This may be related to the effect of the QGSJS decoction intervention in improving body weight, glucose and lipid metabolism, and INS sensitivity in db/db mice [51, 52].

## 5. Conclusion

By using network pharmacology, we identified the core components of QGSJS decoction (quercetin, kaempferol, and isorhamnetin) and the hub genes (IL6, NFKBIA, HIF1A, and VEGFA) that contribute to its anti-DKD effects. The biological mechanisms through which these components exert their effects are likely related to the regulation of hypoxic responses. The results indicate that QGSJS decoction primarily inhibits HIF-1*α* through non–oxygen-dependent pathways, thereby mitigating damage associated with sustained hypoxic responses, including abnormal angiogenesis, inflammation, and fibrosis. This mechanism is presumably the primary means through which it protects the kidneys. The effect of QGSJS decoction on the regulation of DKD hypoxic responses is the result of direct or indirect synergistic interactions with multiple targets and pathways. Further research is required to elucidate its effects on specific pathway targets.

## Figures and Tables

**Table 1:** Active components screened by TCMSP database.

**Mol ID**	**Molecule name**	**OB (%)**	**DL**	**Source**
MOL000398	Isoflavanone	109.99	0.3	Huangqi
MOL000378	7-O-Methylisomucronulatol	74.69	0.3	Huangqi
MOL000392	Formononetin	69.67	0.21	Huangqi
MOL000433	FA	68.96	0.71	Huangqi
MOL000438	(3R)-3-(2-Hydroxy-3,4-dimethoxyphenyl)chroman-7-ol	67.67	0.26	Huangqi
MOL000380	(6aR,11aR)-9,10-Dimethoxy-6a,11a-dihydro-6H-benzofurano[3,2-c]chromen-3-ol	64.26	0.42	Huangqi
MOL000211	Mairin	55.38	0.78	Huangqi
MOL000371	3,9-di-O-methylnissolin	53.74	0.48	Huangqi
MOL000239	Jaranol	50.83	0.29	Huangqi
MOL000354	Isorhamnetin	49.6	0.31	Huangqi
MOL000439	Isomucronulatol-7,2⁣′-di-O-glucosiole	49.28	0.62	Huangqi
MOL000417	Calycosin	47.75	0.24	Huangqi
MOL000098	Quercetin	46.43	0.28	Huangqi
MOL000422	Kaempferol	41.88	0.24	Huangqi
MOL000374	5⁣′-Hydroxyiso-muronulatol-2⁣′,5⁣′-di-O-glucoside	41.72	0.69	Huangqi
MOL000442	1,7-Dihydroxy-3,9-dimethoxy pterocarpene	39.05	0.48	Huangqi
MOL000296	Hederagenin	36.91	0.75	Huangqi
MOL000379	9,10-Dimethoxypterocarpan-3-O-*β*-D-glucoside	36.74	0.92	Huangqi
MOL000033	(3S,8S,9S,10R,13R,14S,17R)-10,13-Dimethyl-17-[(2R,5S)-5-propan-2-yloctan-2-yl]-2,3,4,7,8,9,11,12,14,15,16,17-dodecahydro-1H-cyclopenta[a]phenanthren-3-ol	36.23	0.78	Huangqi
MOL000387	Bifendate	31.1	0.67	Huangqi
MOL000449	Stigmasterol	43.83	0.76	Danggui
MOL000358	Beta-sitosterol	36.91	0.75	Danggui
MOL000471	Aloe-emodin	83.38	0.24	Dahuang
MOL002293	Sennoside D_qt	61.06	0.61	Dahuang
MOL002235	Eupatin	50.8	0.41	Dahuang
MOL002276	Sennoside E_qt	50.69	0.61	Dahuang
MOL000096	(-)-Catechin	49.68	0.24	Dahuang
MOL002251	Mutatochrome	48.64	0.61	Dahuang
MOL002268	Rhein	47.07	0.28	Dahuang
MOL002281	Toralactone	46.46	0.24	Dahuang
MOL002288	Emodin-1-O-beta-D-glucopyranoside	44.81	0.8	Dahuang
MOL002280	Torachrysone-8-O-beta-D-(6⁣′-oxayl)-glucoside	43.02	0.74	Dahuang
MOL002259	Physcion diglucoside	41.65	0.63	Dahuang
MOL000358	Beta-sitosterol	36.91	0.75	Dahuang
MOL002297	Daucosterol_qt	35.89	0.7	Dahuang
MOL002303	Palmidin A	32.45	0.65	Dahuang
MOL002260	Procyanidin B-5,3⁣′-O-gallate	31.99	0.32	Dahuang
MOL000554	Gallic acid-3-O-(6⁣′-O-galloyl)-glucoside	30.25	0.67	Dahuang
MOL000449	Stigmasterol	43.83	0.76	Jianghuang
MOL000953	CLR	37.87	0.68	Jianghuang

**Table 2:** Active components screened by chemical database and SwissADME.

**CAS number**	**Molecule name**	**GI absorption**	**Drug-likeness**					**Source**
			**Lipinski**	**Ghose**	**Veber**	**Egan**	**Muegge**	
4431-01-0	Ligustilide	High	Yes	Yes	Yes	Yes	No	Danggui
89354-45-0	Riligustilide	High	Yes	Yes	Yes	Yes	Yes	Danggui
1135-24-6	Ferulic acid	High	Yes	Yes	Yes	Yes	No	Danggui
499-75-2	Carvacrol	High	Yes	No	Yes	Yes	No	Danggui
458-37-7	Curcumin	High	Yes	No	Yes	Yes	Yes	Jianghuang
22608-11-3	Demethoxycurcumin	High	Yes	No	Yes	Yes	Yes	Jianghuang
160096-59-3	Dimethoxycurcumin	High	Yes	No	Yes	Yes	Yes	Jianghuang
63-68-3	Methionine	High	Yes	No	Yes	Yes	No	Chantui
529-69-1	Isoxanthopterin	High	Yes	No	Yes	Yes	No	Chantui
13594-27-9	Beauverilide A	High	Yes	No	No	Yes	No	Jiangcan
54278-73-8	Bassianin	High	Yes	Yes	Yes	Yes	Yes	Jiangcan
484-78-6	3-Hydroxy kynurenine	High	Yes	Yes	Yes	Yes	No	Jiangcan
53823-15-7	Tenellin	High	Yes	Yes	Yes	Yes	Yes	Jiangcan
373-49-9	Palmitoleic acid	High	Yes	Yes	No	Yes	No	Jiangcan
5289-74-7	Ecdysterone	High	Yes	No	Yes	No	No	Jiangcan
9001-06-3	Chitinase	High	Yes	Yes	Yes	Yes	Yes	Jiangcan
520-18-3	Kaempferol	High	Yes	Yes	Yes	Yes	Yes	Jiangcan
117-39-5	Quercetin	High	Yes	Yes	Yes	Yes	Yes	Jiangcan

**Table 3:** Efficacy of QGSJS decoction in intervening DKD (*n* = 6).

**Item**	**db/m**	**db/db**	**db/db + QGSJS**
FBG (mmol/L)	8.73 ± 2.84	29.92 ± 3.33^△△△^	28.93 ± 2.02
GSP (mmol/L)	4.21 ± 0.24	6.37 ± 0.42^△△△^	5.43 ± 0.72^∗∗^
INS(*μ*U/mL)	29.91 ± 2.37	29.36 ± 5.96	20.93 ± 3.45^∗∗^
HOMA-IR	11.63 ± 3.93	39.36 ± 10.81^△△△^	26.85 ± 4.53^∗∗^
HOMA-IS	0.093 ± 0.026	0.026 ± 0.007^△△^	0.038 ± 0.006^∗^
Cr (*μ*mol/L)	207.92 ± 12.70	228.55 ± 21.96^△△△^	171.43 ± 5.5^∗∗^
BUN (mmol/L)	4.21 ± 0.23	6.30 ± 0.52^△△△^	5.28 ± 0.59^∗∗^
TG (mmol/L)	0.75 ± 0.15	1.46 ± 0.30^△△^	1.12 ± 0.15
TC (mmol/L)	1.75 ± 0.14	2.93 ± 0.59^△△△^	2.10 ± 0.39^∗∗^

*Note:* Compared to the db/db group: ⁣^∗^*p* < 0.05 and ⁣^∗∗^*p* < 0.01; compared to the db/m group: ^△△△^*p* < 0.001 and ^△△^*p* < 0.01.

## Data Availability

The datasets used and/or analyzed during the current study are available from the corresponding authors upon reasonable request.

## Nomenclature

QGSJS: Qi-Gui-Sheng-Jiang-San
DGBXT: Dang-Gui-Bu-Xue-Tang
SJS: Sheng-Jiang-San
DKD: diabetic kidney disease
TCMSP: Traditional Chinese Medicines Systems Pharmacology database
OMIM: Online Mendelian Inheritance in Man
DL: drug-likeness
OB: oral bioavailability
ADME: absorption, distribution, metabolism, and excretion
PPI: protein–protein interaction
CC: cellular component
MF: molecular function
BP: biological process
GO: Gene Ontology
KEGG: Kyoto Encyclopedia of Genes and Genomes
HIF-1*α*: hypoxia-inducible factor 1-alpha
STAT3: signal transducer and activator of transcription 3
VEGFR: vascular endothelial growth factor receptor
VEGF: vascular endothelial growth factor
NF-*κ*B: nuclear factor kappa-B
PHD2: prolyl hydroxylase domain containing 2
PI3K/Akt: phosphoinositide 3-kinase/Akt
